# Amorphous metal oxide mixtures for high-*Q* integrated nonlinear photonics

**DOI:** 10.1038/s42005-026-02665-w

**Published:** 2026-05-07

**Authors:** Alexa R. Carollo, Atasi Dan, Haixin Liu, David R. Carlson, Jennifer A. Black, Scott B. Papp

**Affiliations:** 1 Time and Frequency Division, National Institute of Standards and Technology, Boulder, CO USA; 2 Department of Physics, University of Colorado, Boulder, CO USA; 3 Octave Photonics, Louisville, CO USA; 4 Present Address: Vescent Photonics, Golden, CO USA

**Keywords:** Nonlinear optics, Nonlinear optics, Microresonators

## Abstract

Choice of material is ubiquitous in integrated photonics to design device properties, whereas changing material composition is much less common. With phase matching as an additional objective, constraints in depositing and patterning thin films limit the use of integrated nonlinear photonics to only select materials. Here, we explore an amorphous metal oxide mixture of titania (TiO_2_) and tantala (Ta_2_O_5_) in which material composition is a tool to enhance and customize photonics properties. In particular, the inclusion of titania reduces the oxygen-defect density in a tantala film, while maintaining a comparable Kerr nonlinear index. With ion-beam sputtering at room temperature, we deposit a thick, ultralow-loss titania-tantala film, and we nanopattern it to create microresonator frequency combs. Titania-tantala microresonators offer lower loss, higher index of refraction, and reduced optical absorption and photorefractive effects. Specifically, the titania-tantala mixture enables microresonator quality factor up to 10^7^ and a direct factor of 1.7 reduction in optical absorption. This, along with its large transparency window, high nonlinearity, and low processing temperature requirement, positions titania-tantala as a leading material for integrated nonlinear photonics. Our work demonstrates that composition in metal-oxide mixtures is a design parameter alongside the nanofabrication process and photonics design for integrated nonlinear photonics.

## Abstract (web summary)

Laser wavelength conversion in integrated platforms requires nonlinear materials with low loss, but available materials are limited due to phase matching and fabrication constraints. The authors explore an amorphous mixture of tantala (Ta_2_O_5_) and titania (TiO_2_), showing that film composition can be tailored to enhance performance and compatibility.

## Introduction

Integrated photonics offers various high-value functions, including dense and versatile circuits^1^, access to modulation^2,3^ and photodetection^4^, integration with microelectronics^5^, and laser gain with heterogeneous integration^6^. To take advantage of the unprecedented potential of integrated-photonics systems, low optical loss is critical for distributing light among complex circuits and between interfaces, placing constraints on photonic-material compatibility. Nonlinearity is an emerging function in integrated photonics that enables conversion from one wavelength to another and generation of quantum-based light states. Low loss and small mode volume are key to access nonlinearity, subject to control of group-velocity dispersion. The development of integrated nonlinear photonic microsystems drives innovation in low-loss materials, especially in improving their compatibility with integration and device geometric constraints, to open up opportunities in extreme-capacity data transmission^7–12^, advanced computing architectures^13,14^, quantum sensing^15–21^, and signal generation^22–24^. Numerous discrete nonlinear photonics materials have been explored, and realizing fully functional platforms so far requires the use of different materials to overcome limitations in, for example, transparency or processing requirements.

To discern the tradeoffs between fabrication compatibility of materials and low optical loss, we survey the properties of different material platforms. Stoichiometric silicon nitride (Si_3_N_4_, or SiN) is an exemplary material for integrated nonlinear photonics, owing to relatively high refractive index for device design and group-velocity dispersion engineering, relatively high nonlinear coefficient, access to microresonator intrinsic quality factor, *Q*_i_, above 10 million, and high power handling without photorefraction and multi-photon absorption^25–31^. However, the end-to-end process with low-pressure chemical-vapor-deposited (LPCVD) SiN requires high temperature 1200 °C annealing and the films have substantial tensile stress, compromising integration compatibility with other materials. For example, integration of SiN with thin film lithium niobate requires wafer bonding^32^ instead of direct deposition to avoid exposing the lithium niobate to high temperatures that degrade the material. Other formulations and deposition techniques, e.g., sputtering^33^, plasma-enhanced CVD^34^, and hot-wire CVD^35^, lower the temperature requirement and enhance integration compatibility, but result in higher absorption that degrades microresonator *Q*_i_. Crystalline materials like lithium niobate (LN) and semiconductors like gallium arsenide (GaAs) offer a wide variety of functionalities, but require complex wafer bonding and processing temperature constraints. The trend of such tradeoffs is common in integrated photonics, especially in gaining access to nonlinearity.

While adjusting material composition is not common with ultralow-loss SiN photonics, semiconductor materials like GaAs and crystalline materials like LN are often customized with dopants. GaAs and its alloys make up an exceptional platform for laser gain, both in discrete epitaxial structures and through heterogeneous integration of epitaxial waveguide layers to low-loss passive and nonlinear materials^36^. Furthermore, doping LN with magnesium oxide offers lower optical losses in the visible and reduced photorefractive effects for improved stability of its optical properties over undoped LN^37^. On the other hand, amorphous metal oxide mixtures form a class of low-loss dielectric materials that offers an exceptional range of variation in composition^38^. There are numerous metal oxides with unique optical properties, including tantala (Ta_2_O_5_)^39–41^, germanium oxide^42^, tungsten trioxide^43^, and barium titanate^44^. Compared to SiN, tantala offers a wider transparency window^45^, a larger Kerr coefficient, and a lower processing temperature requirement. With metal oxides, sputtering deposition at low temperature and on arbitrary substrates is common^46,47^.

Here, we show that material composition in metal-oxide mixtures is a viable design parameter for optimizing integrated nonlinear photonics. Specifically, we explore an amorphous film that is a mixture of two metal oxides, titania (TiO_2_) and tantala, motivated by use of this mixture in LIGO mirrors due to lower mechanical loss^48^. Titania-tantala features an intrinsic reduction in oxygen-vacancy defects, resulting in reduced optical absorption and photorefractive effects while maintaining a high nonlinear index. We demonstrate a robust, wafer-scale, CMOS-compatible nanofabrication process flow with all steps operated at < 500 °C that realizes microresonators with *Q*_i_ up to 1 × 10^7^. Quality factors of this magnitude are not readily achievable in other materials, including pure tantala, with comparable processing temperatures. Our results introduce a method for controlling material properties, and we demonstrate progress towards overcoming the high-temperature requirements of ultralow-loss platforms. This work has the potential to enable fully integrated on-chip systems for future applications in optical frequency metrology, precision spectroscopy, and quantum sensing.

## Results and discussion

### Titania-tantala microresonator *Q*_i_ and comparison to existing platforms

**Fig. 1:**
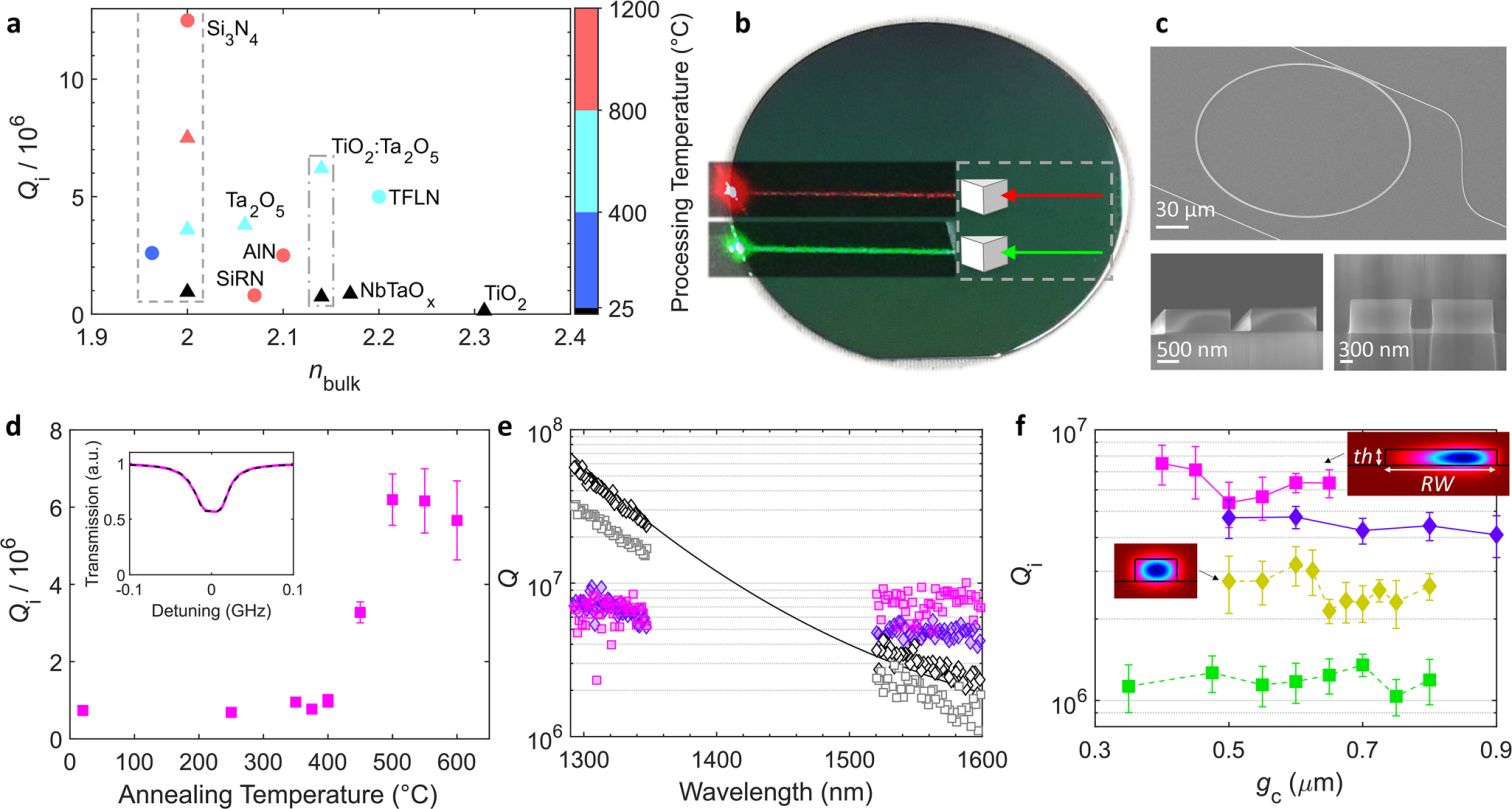
Characterization of titania-tantala films and microresonators. **a** Intrinsic *Q* and *n*_bulk_ at 1550 nm, comparing different photonics materials with triangles for sputtered films and point color for processing temperature. Repeated points indicate the sensitivity of *Q*_i_ to fabrication and processing conditions. Increased *Q*_i_ is reported for Si_3_N_4_ with nonstandard fabrication approaches^51,68^, which we indicate by the open box. We label titania-tantala as TiO_2_:Ta_2_O_5_, pure tantala as Ta_2_O_5_^39,41^, silicon nitride as Si_3_N_4_^33,34,51^, silicon-rich silicon nitride as SiRN^52^, thin-film lithium niobate as TFLN^54^, niobium-tantalum oxide as NbTaO_x_^46^, aluminum nitride as AlN^53^, and pure titania as TiO_2_^55,56^. **b** An oxidized silicon wafer coated with a titania-tantala film. The overlays illustrate prism coupling of laser light into the film at wavelengths of 637 nm (top) and 520 nm (bottom). **c** Scanning-electron microscope (SEM) image of a microresonator (top), and cross sections of two waveguides separated by 0.500 μm with air (bottom left) and oxide cladding (bottom right). **d** *Q*_i_ as a function of post-fabrication annealing temperature. Error bars are the standard deviation of *Q*_i_ of resonance modes across the 1520–1600 nm wavelength range for a given device. Inset: mode transmission spectrum (pink) and its fit (black). **e** *Q*_i_ (colors) and *Q*_c_ (grayscale) as a function of wavelength for devices with different claddings. Squares indicate air cladding and diamonds indicate oxide cladding. The black line is an FDTD simulation of *Q*_c_, scaled by 0.6, for the oxide clad device. **f** *Q*_i_ as a function of *g*_c_ for air clad (squares) and oxide clad (diamonds) devices with different RW. Points connected by solid and dashed lines are RW = 4 μm and RW = 1.5 μm, respectively. Error bars are the standard deviation of *Q*_i_ of resonance modes across the 1520–1600 nm wavelength range for each device. Insets: simulated electric field amplitude.

The principal advantage of titania-tantala for integrated nonlinear photonics is the combination of moderately high index of refraction, a modest process temperature requirement, and record high quality factor. In this work, we begin with titania-tantala films deposited on the amorphous SiO_2_ layer of a 3-inch thermally oxidized silicon wafer. This way, we achieve sufficient index contrast between our films and the substrate for high confinement of light in waveguides and microresonators. To create our film mixtures, we co-sputter titanium and tantalum metal targets via ion-beam sputtering at room temperature. We design our films with a 0.26 volume fraction of titania, meaning that 26% of the film is titania and the remaining 74% is tantala. From systematic studies of loss as a function of composition in mirror coatings for LIGO, this composition should have close to the lowest defect density out of a host of metal oxide mixtures with different species and concentrations^38,49,50^. To test this in the context of integrated nonlinear photonics, we also explore a mixture of tantala and 13% titania. Indeed, its performance is in between that of pure tantala and the film with higher titania content, based on *Q*_i_ and resistance to photorefraction. While we do not focus on the intermediate concentration in this work, it demonstrates the versatility of the tantala platform at producing high-quality amorphous films with custom composition. We put the properties of titania-tantala into context by plotting microresonator *Q*_i_ versus bulk index of refraction, *n*_bulk_, for several comparable materials in the 1550 nm wavelength region; see Fig. 1a and refs. ^25,33,34,41,46,51–56^. Deposition technique and processing temperature are the factors that control the integration of nonlinear materials. We define processing temperature as the highest of the reported deposition or annealing temperature. To date, silicon nitride provides the highest *Q*_i_, but only when annealed at 1200 °C. Titania-tantala is promising because the temperature requirement to achieve high *Q*_i_ is much more favorable. Even compared to pure tantala, titania-tantala offers the highest *Q*_i_ of materials processed at moderate temperatures (≤500 °C). The effect of annealing metal oxide mixtures is primarily a reduction in absorptive oxygen-vacancy defects. Apparently, this is obtained at a much lower temperature than that required to remove hydrogen impurities in silicon nitride. Even without annealing, the intrinsic defect density of titania-tantala is low enough to provide a *Q*_i_ of 7 × 10^5^ with full room temperature processing. Moreover, unannealed titania-tantala offers low visible-band absorption. We couple visible laser light into the thin film with a prism and quantify propagation loss; see Fig. 1b. We use a Metricon prism coupler system to measure scattered laser light at different positions as it propagates across the wafer. The data is fit to an exponential curve to extract propagation loss. Our measurements approach the instrument limit, achieving propagation loss < 0.2 dB cm^−1^. This flexibility in processing temperature and the ability to fabricate materials with custom composition are unique advantages of sputtered metal oxide films.

To explore integrated photonics devices in titania-tantala films, we fabricate microresonators with a typical CMOS process, including electro-beam lithography, reactive ion etching, silicon dioxide (SiO_2_, hereafter oxide) cladding deposition, and thermal annealing. This yields microresonators and integrated waveguide couplers; see Fig. 1c. The primary consideration in designing microresonators is for nonlinear optics, so we choose the titania-tantala film thickness th = 0.570 μm for air clad devices and th = 0.800 μm for oxide clad devices. These thicknesses enable fine-tuning of the group-velocity dispersion (GVD) throughout the anomalous and normal regimes by changing the ring width (RW) and ring radius (RR) of resonators. For external coupling of microresonators, we use pulley couplers that are optimized for broadband and selective coupling of the TE0 mode^57^. To assess the etch quality, we fabricate test structures and take SEM images of their cross sections. In both air and oxide clad devices, we observe slight deviations from the designed rectangular profiles due to low etch selectivity. Apparently, the inclusion of titania reduces the etch selectivity, which could contribute to sidewall roughness and degradation of *Q*_i_ below the true material limit. For a direct comparison of the two materials, we use the same fabrication procedure that is originally developed for pure tantala. Future work could include optimizing the etch parameters (e.g., precursor flow rate, RF power, temperature) specifically for titania-tantala.

Since annealing is critical to increase *Q*_i_ in titania-tantala resonators, we characterize its temperature dependence, while maintaining appropriate annealing time and gas composition; see Fig. 1d. In metal oxide films, oxygen-vacancy defects form during film deposition, exposure to vacuum, and exposure to UV radiation. The defects are absorptive and reduce *Q*_i_ below the true material limit. Their structure and distribution amongst the surface and bulk is widely studied to control and optimize electrical and optical properties. For example, a common defect in amorphous films is TaO_2_, which is oxygen deficient compared to tantala (Ta_2_O_5_)^58^. As a way to increase the oxygen content, the films are annealed in air. This drives diffusion and chemical reactions with oxygen that reduce the defect density. We explore the effects of annealing in air clad microresonators with RW = 4 μm and RR = 100 μm. Indeed, our experiments reveal temperature-dependent behavior of *Q*_i_ that reflects changes in material absorption. We find a rapid increase in *Q*_i_ up to (3.3 ± 0.3) × 10^6^ by annealing at 450 °C. This sharp transition indicates that oxygen has ample energy to diffuse into the film and react with oxygen-vacancy defects. The effect of annealing saturates above 500 °C, with an average *Q*_i_ = (6.2 ± 0.7) × 10^6^. Our results show that titania-tantala has the flexibility to be used across a wide range of temperatures, with maximum *Q*_i_ approaching the material absorption limit when annealed up to 600 °C. An important feature of titania-tantala is that it remains crack-free across this temperature range. We anticipate crystallization of the film beyond 600–700 °C^49^, which has not been explored with the production of tantala photonics because of the likelihood of cracking.

With our optimized annealing process, we explore *Q*_i_ in titania-tantala microresonators versus wavelength and the choice of either air or oxide cladding; see Fig. 1e. Here, a number of factors influence *Q*_i_, predominantly absorption in the titania-tantala and oxide cladding materials. The large RW should substantially reduce scattering from the etched surfaces for both the 1300 and 1550 nm wavelength bands. The external coupling rate of the devices shifts between the two bands; hence, we plot the corresponding coupling quality factor, *Q*_c_, of the *Q*_i_ measurements as open points. An FDTD (finite-difference time-domain) calculation of *Q*_c_ with cladding explains the measured results within a scale factor of 0.6. In the 1300 nm band, we expect the oxide cladding to have low absorption^59^. Comparing air and oxide clad *Q*_i_ in this wavelength region allows us to assess potential damage to the titania-tantala layer induced by the oxide deposition process. We do not observe a significant difference; in fact, we achieve high *Q*_i_ up to 9.5 × 10^6^ in the oxide clad device. Hence, the film’s robustness and our annealing process can ensure low loss, even after the oxide deposition step. In the 1550 nm band, where the mode is larger and extends further outside the material, the results are different. We achieve *Q*_i_ up to 1 × 10^7^ in the air clad device, but the *Q*_i_ for the oxide clad resonators is lower. Absorption in the oxide cladding is the likely cause.

To further investigate the factors that limit *Q*_i_, we systematically vary the microresonator device geometry to influence the relative impact of absorption and scattering losses; see Fig. 1f. We have the capability to widely vary the external coupling rate through changes in the resonator-waveguide gap distance, *g*_c_. This is an important factor in diagnosing loss mechanisms because it controls *Q*_c_. In our largest RW air clad microresonators, we vary *g*_c_ from 0.4 to 0.65 μm. This induces a change in *Q*_c_ from 2 × 10^6^ to 6 × 10^7^, while maintaining a constant *Q*_i_ near 6 × 10^6^. The *Q*_i_ is unchanged over this range of *g*_c_ for several settings of RW, demonstrating control of the coupling rate without introducing parasitic coupling loss. As we decrease the cross-sectional dimension of the ring, we reveal a transition from absorption-limited to scattering-limited *Q*_i_. Decreasing the RW to 1.5 μm causes the mode profile of the propagating light to extend further into the sidewalls, which have rough surfaces from the etch process. The rough surfaces induce scattering, which degrades *Q*_i_ below the absorption limit.

### Effect of material composition on absorption

**Fig. 2:**
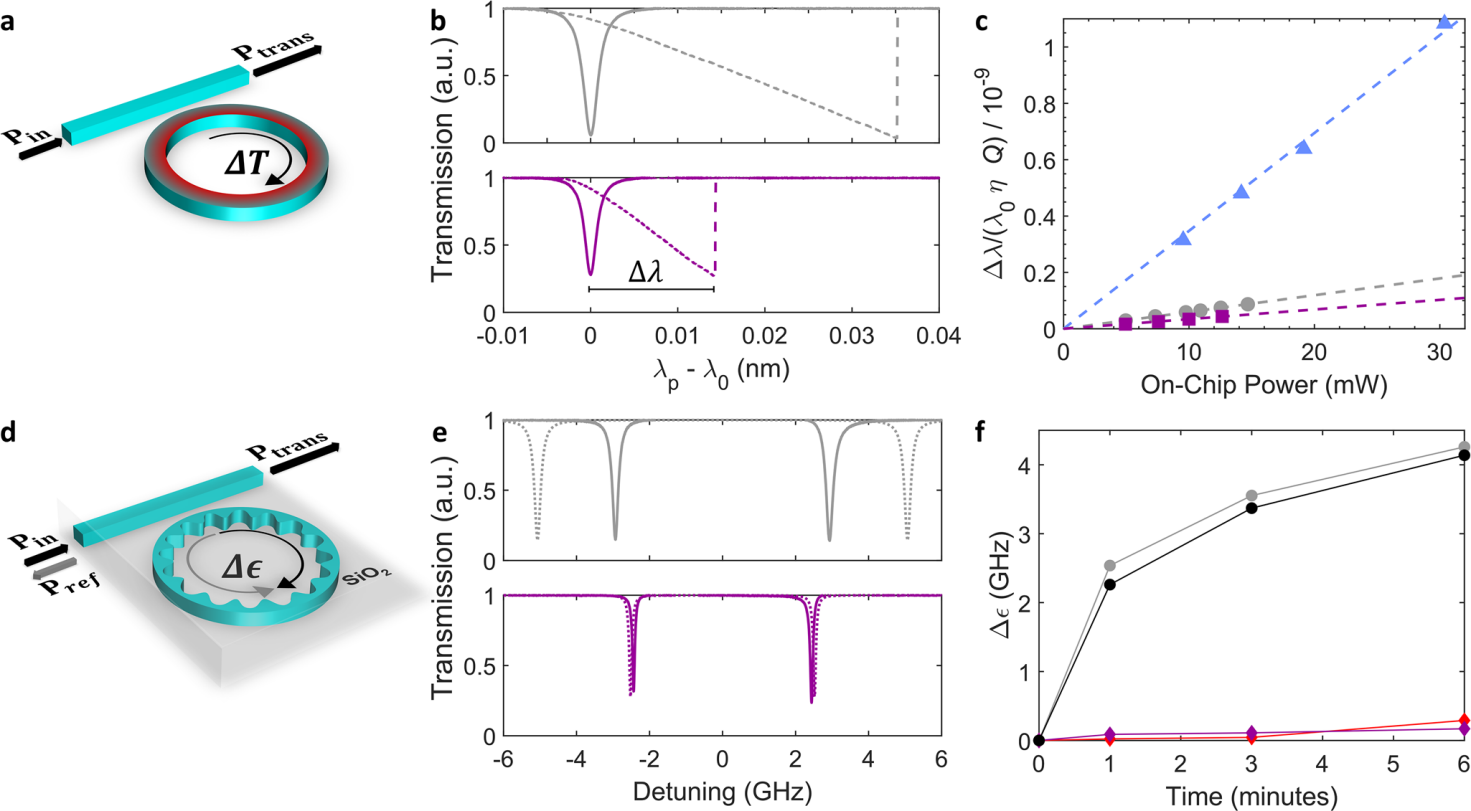
Exploring defect density in titania-tantala microresonators. **a** Microresonator with power-dependent absorption. *P*_in_: input power, *P*_trans_: transmitted power, Δ*T*: change in temperature. **b** Power-dependent transmission lineshapes for tantala (gray) and titania-tantala (magenta) as a function of pump laser detuning, *λ*_p_ − *λ*_0_. Solid lines: low power measurement. Dashed lines: high power measurement with ≈10 mW of on-chip power. The resonance shift due to heating, Δ*λ*, is indicated on the plot. **c** Δ*λ* (scaled) as a function of on-chip power for annealed tantala (gray circles), annealed titania-tantala (magenta squares), and unannealed titania-tantala (blue triangles), along with linear fits (dashed lines). **d** Illustration of an oxide clad PhCR. *P*_in_: input power, *P*_trans_: transmitted power, *P*_ref_: reflected power, Δ*ϵ*: change in mode splitting. **e** Measured transmission spectrum of a PhCR before pumping (solid lines). Dashed lines represent the change in transmission spectrum after pumping with 100 mW of on-chip power for 6 min. Oxide clad tantala is shown in gray, and oxide clad titania-tantala is shown in magenta. **f** Δ*ϵ* after pumping with 100 mW on-chip power as a function of pumping time for two different tantala (gray and black circles) and titania-tantala (magenta and red diamonds) PhCR devices.

The central advantage of changing composition in a metal oxide mixture is to enhance device properties without otherwise modifying the fabrication process. Its high *Q*_i_ suggests that titania-tantala has a lower defect density than pure tantala, but since *Q*_i_ is affected by scattering introduced during fabrication, it is not a direct measurement of absorption. Here, we directly test for reductions in optical absorption and effects like photorefraction that depend on defect density. For optical absorption, we measure the quality factor associated with absorption, *Q*_abs_, by microresonator thermal bistability. We directly compare titania-tantala and pure tantala microresonators fabricated under the same conditions to characterize their difference in defect density. Our thermal bistability measurements follow the canonical model of Carmon et al.^60^. A microresonator mode resonance wavelength (*λ*_0_) shifts with a change in temperature (Δ*T*) as

1 $${\lambda }_{{\rm{r}}}(\Delta T)={\lambda }_{0}(1+a\Delta T),$$

where *a* = *α* + (d*n*/d*T*)/*n*_0_ is the temperature coefficient, containing the thermal expansion coefficient (*α*), the thermo-optic coefficient (d*n*/d*T*), and index of refraction (*n*_0_). In our experiments, Δ*T* is induced by absorption of the pump laser in the material, as illustrated in Fig. 2a. As the pump laser wavelength (*λ*_p_) sweeps from lower to higher wavelength, the material dynamically heats and the mode resonance thermally broadens; see Fig. 2b. Notably, the shift in resonance wavelength (Δ*λ* = *λ*_r_ − *λ*_0_) is larger in tantala than in titania-tantala, which indicates higher absorption and defect density in tantala.

We systematically measure Δ*λ* at various pump powers to extract *Q*_abs_ for three different microresonators; see Fig. 2c. Our primary goal is to compare absorption in titania-tantala and tantala, with a focus on devices annealed at 500 °C for the highest *Q*_i_. By also comparing with an unannealed titania-tantala microresonator, we quantify the underlying absorption changes induced by annealing. At steady state and when *λ*_p_ is on resonance with *λ*_r_ (at the transmission minimum), Δ*T* = *IηQ*/(*Q*_abs_*k*), where *I* is the on-chip pump power, *η* is the coupling efficiency (*η* = (1 + 1/*K*)^−1^), *Q* is the total quality factor, and *k* is the thermal conductivity of the mode volume with the surroundings. The coupling parameter, *K*, is defined as *K* ≡ *Q*_i_/*Q*_c_. This relationship, and the relationship between Δ*T* and Δ*λ* in Eq. 1, allows us to directly extract information about *Q*_abs_. With corrections for differences in microresonators, we observe a linear dependence of our scaled Δ*λ* with *I*, with annealed titania-tantala yielding the smallest slope. The slope encompasses the remaining unknown parameters, *a*/(*Q*_abs_*k*). To simplify our analysis, we assume that the thermal parameters, *a* and *k*, are the same for all three films. With these assumptions, we can directly compare *Q*_abs_. As expected from the drastic improvement in titania-tantala’s *Q*_i_ with annealing, we find that *Q*_abs_ increases by a factor of 10 with annealing. This is consistent with the > 8 times improvement in *Q*_i_, validating our assumptions in this experiment. Compared to annealed tantala, annealed titania-tantala has a 1.7 times higher *Q*_abs_. This is also consistent with our *Q*_i_ measurements, but this experiment provides direct evidence that metal oxide mixtures support intrinsically low defect density.

### Effect of material composition on photorefraction

Photorefraction, an effect in which the material index of refraction changes with optical intensity, is a consequence of high defect density. In tantala microresonators, it occurs due to charge redistribution and build-up in defect sites. The effect induces coherent backscattering and optical interference of a large enough magnitude that can hinder GVD phase matching and reduce efficiency. Coherent backscattering causes a transient split of the pump resonance. The magnitude of the induced splitting depends on pumping time, *t*, as

2 $$\epsilon (t)={\epsilon }_{0}+\xi (1-{{\rm{e}}}^{-t/{\tau }_{{\rm{p}}}})| {\beta }_{+}^{* }{\beta }_{-}|$$

where *ϵ*_0_ is the static splitting, *ξ* is a material parameter, *τ*_p_ is the time constant, and $| {\beta }_{+}^{* }{\beta }_{-}|$ is the intensity of the standing wave generated by the forward (*β*_+_) and backward (*β*_−_) propagating fields in the resonator^61^. We assume that *ξ*, which controls the magnitude of the time-dependent part, is related to defect density in metal oxides. A higher defect density should have a larger photorefractive effect because there are more defect sites available for charges to accumulate.

In a photonic-crystal microresonator (PhCR), we intentionally inscribe a modulation of the RW during device fabrication; see Fig. 2d. The modulation induces coherent backscattering and generates a band gap with splitting of *ϵ*_0_, which facilitates soliton microcomb generation^62^. However, the standing wave created within the resonator is enhanced by the photorefractive effect, increasing the mode splitting by Δ*ϵ* = *ϵ* − *ϵ*_0_ from the designed value. Oxide-clad tantala devices, in particular, suffer from significant photorefractive splitting; see Fig. 2e. After pumping the PhCR mode with *I* = 100 mW for 6 min, the Δ*ϵ* is 4.2 GHz. On the contrary, oxide-clad titania-tantala is much more robust. The Δ*ϵ* is only 0.17 GHz, which is 25 times less than tantala. To understand the dynamics, we investigate Δ*ϵ* as a function of pumping time; see Fig. 2f. For both tantala PhCRs (gray and black traces), Δ*ϵ* exceeds 2 GHz after pumping for only 1 min, and approaches saturation by 6 minutes. For both titania-tantala PhCRs (red and magenta traces), Δ*ϵ* remains below 0.3 GHz at all times. The saturation dynamics are less apparent, perhaps due to the small Δ*ϵ*. Since the photorefractive splitting is significantly smaller in titania-tantala, this indicates that its defect density is intrinsically lower than tantala.

### Nonlinearity in titania-tantala integrated devices

Our characterization of the titania-tantala metal oxide mixture indicates that composition does offer the opportunity to enhance and customize integrated photonics properties. Here, we measure the threshold and operation of nonlinear wavelength conversion in titania-tantala to assess the Kerr nonlinear index of the mixture in comparison with pure tantala. To measure the Kerr nonlinear index, we explore the threshold power for microresonator optical parametric oscillation (OPO) and the generation of supercontinuum from an input modelocked laser.

**Fig. 3:**
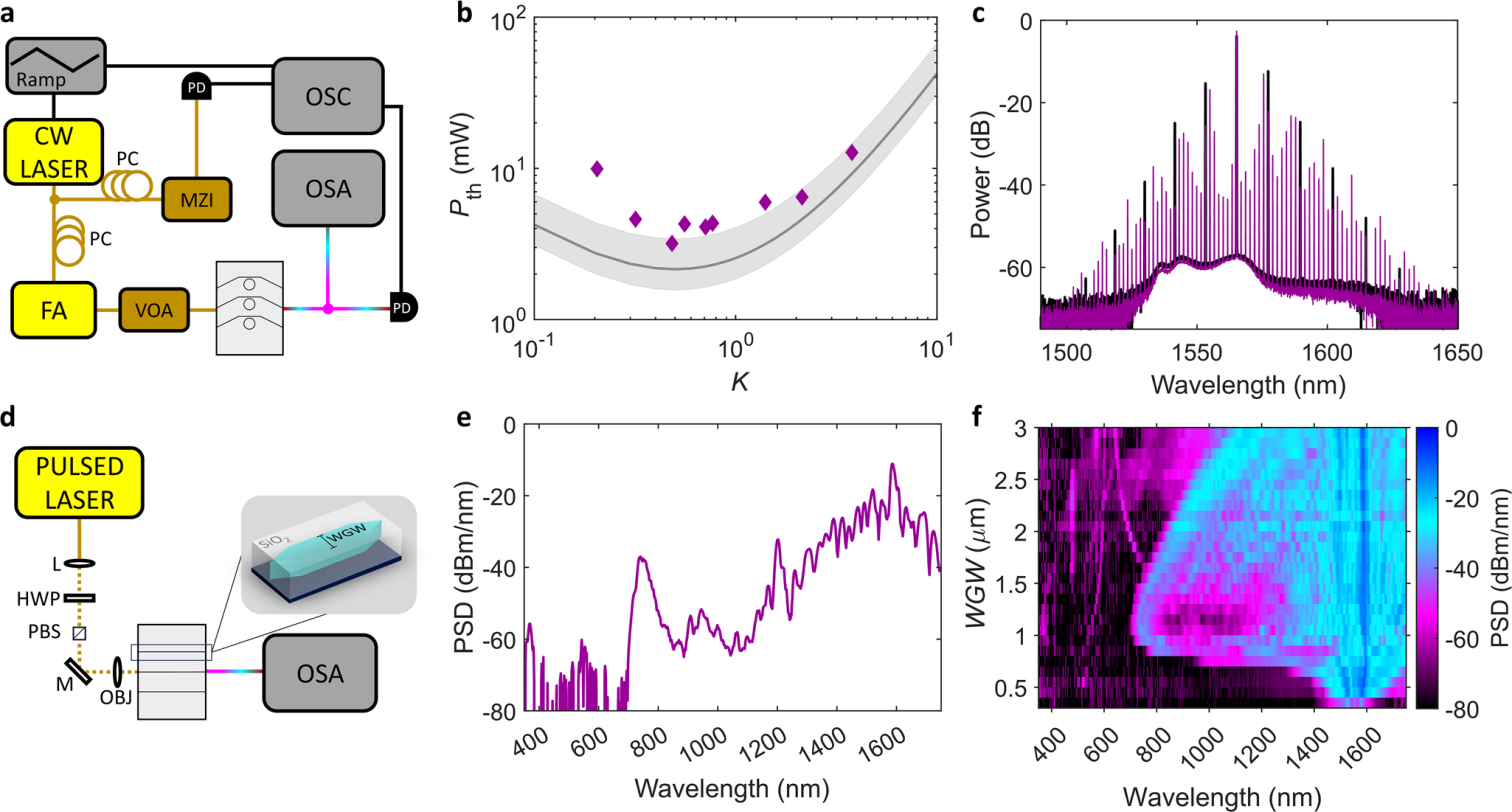
Nonlinearity in titania-tantala. **a** Experimental setup for generating OPO and microcombs. CW: continuous wave, PC: polarization control, FA: fiber amplifier, MZI: Mach–Zehnder interferometer, PD: photodiode, VOA: variable optical attenuator, OSC: oscilloscope, OSA: optical spectrum analyzer. **b** Experimentally measured threshold power, *P*_th_, for a set of oxide clad microresonators with different coupling parameters, *K* (magenta diamonds), along with a calculation using *n*_2_ of tantala (gray line). We use the uncertainty in *n*_2_ to calculate upper and lower bounds of *P*_th_ (gray band). **c** Microcomb spectra from pumping a microresonator with 11 mW (black) and 17 mW (magenta) on-chip power, scaled relative to pump power (0 dB). **d** Experimental setup for generating supercontinuum. L: lens, HWP: half-waveplate, PBS: polarizing beamsplitter, M: mirror, OBJ: objective lens. **e** Supercontinuum spectrum generated from pumping a WGW = 1 μm oxide clad device with 0.6 nJ pulse energy. **f** Contour plot of supercontinuum spectra as a function of WGW.

To generate OPO in titania-tantala microresonators, we select devices of a fixed resonator geometry, and we use variation in the external coupling to systemically study the OPO threshold power. To satisfy the phase-matching conditions for OPO generation, we design the geometric parameters to support anomalous GVD; see Supplementary Note 1. For a microresonator with oxide cladding, we achieve this with a RR = 100 μm and RW = 1.5 μm. We pump the microresonator with a continuous-wave (CW) laser, using the experimental arrangement in Fig. 3a, and we record the pump power for OPO initiation. The OPO threshold power depends on the Kerr nonlinear index, *n*_2_, as

3 $${P}_{{\rm{th}}}=\frac{\pi {n}_{0}{\omega }_{0}{A}_{{\rm{eff}}}}{4{n}_{2}{D}_{1}{Q}_{{\rm{i}}}^{2}}\frac{{(K+1)}^{3}}{K}$$

where *ω*_0_ is the optical frequency, *A*_eff_ is the effective mode area, and *D*_1_ is the free spectral range. Indeed, the geometry of the microresonator determines many of the parameters that influence *P*_th_. For a fixed geometry, a way to optimize *P*_th_ is through adjusting the external coupling. We vary *g*_c_ to adjust the coupling parameter, *K* ≡ *Q*_i_/*Q*_c_, and measure its effect on *P*_th_; see Fig. 3b. As predicted from Eq. 3, we observe a dependence on *K*, with a minimum at *K* = 0.5. Our prediction uses the *n*_2_ of tantala, which is (6.2 ± 2.3) × 10^−19^ m^2^ W^−1^^41^. Since our measured values fall near the predicted values across a range of *K*, we estimate that the *n*_2_ of titania-tantala is comparable to tantala. Apparently, the modification to the film composition has a minimal effect on *n*_2_. When pumping above the threshold, we observe cascaded OPO at the onset of microcomb formation; see Fig. 3c. While not a soliton, this microcomb is a useful demonstration of the material’s nonlinearity. We explore soliton generation in PhCRs designed to have normal and anomalous GVD in Supplementary Note 2, confirming that the material can support soliton generation. Since the index of refraction is not significantly altered by the inclusion of titania, we anticipate that titania-tantala should be able to support bright soliton generation and broader spectra with dispersion engineering of the microresonator RR, RW, and th, as demonstrated in pure tantala^39,41^.

To further explore the Kerr nonlinearity of the titania-tantala mixture, we generate supercontinuum in a waveguide and characterize its spectral properties. The high confinement in waveguides naturally allows access to nonlinear processes that broaden the spectrum of an input modelocked laser. We use a 1550 nm band modelocked laser to pump a fully oxide clad titania-tantala waveguide and record its output spectrum by use of the experimental setup in Fig. 3d. We design the waveguide width (WGW) such that the GVD supports a broad supercontinuum; see Fig. 3e. Notably, the power spectral density (PSD) in the 750 nm wavelength region is enhanced by a dispersive wave. The dispersive wave location agrees with simulations of the waveguide dispersion; see Supplementary Note 3. We control the wavelength of the dispersive wave by changing the WGW, as demonstrated in Fig. 3f. As we vary the WGW from 1 to 3 μm, the dispersive wave shifts from 750 to 1100 nm. The input pulse energy required to generate these spectra is 0.6 nJ, which is comparable to tantala^40^. Since *n*_2_ controls the energy required for supercontinuum generation, these results provide further evidence that the nonlinear behavior of the mixture is comparable to pure tantala.

## Conclusions

In conclusion, we have demonstrated a powerful method to engineer material properties for integrated nonlinear photonics by tailoring the composition of amorphous metal oxide films. By introducing titania into tantala, we reduce defect density without altering the low-temperature, CMOS-compatible fabrication process. This composition-driven approach yields record-high quality factors at < 500 °C, significantly lowering absorption and suppressing photorefractive effects, while preserving the high Kerr nonlinearity of tantala. These improvements position titania-tantala as an enabling material for low-loss, nonlinear, and dispersive photonic devices. While our work focuses on titania-tantala for the lowest defect density and high *Q*_i_, there is a myriad of metal oxide mixtures with other species (e.g., Al_2_O_3_, ZnO, Nb_2_O_5_, …) that offer the opportunity to tailor film crystallization temperature, index of refraction, nonlinear index of refraction, and band gap^38,46,49^. These properties have an important role in practical applications, but many of these mixtures have yet to be explored specifically for integrated nonlinear photonics. Since there is a wide range of compositions that produce high-quality amorphous films at room temperature, our work opens up a research avenue for exploring their unique properties in the context of integrated nonlinear photonics. Heterogeneous integration with crystalline substrates is promising, since titania-tantala can be directly deposited on crystalline silicon^38^. Wafer bonding to crystalline GaAs has yet to be investigated, but techniques developed for pure tantala^6^ could be applied to metal oxide mixtures. Looking ahead, this work establishes a pathway to scalable, monolithically integrated photonic systems for next-generation applications in frequency metrology, communications, and quantum technologies.

## Methods

### Material platform comparison

To compare different materials within the context of integrated nonlinear photonics, we explore *Q*_i_ versus *n*_bulk_ and note the required processing temperature. These factors bring together considerations in integrating different photonics materials and achieving phase matching for four-wave mixing. We plot average *Q*_i_ if reported; otherwise, we plot highest *Q*_i_ in Fig. 1a. We plot *Q*_i_, rather than absorption-limited quality factor, because *Q*_i_ contains contributions from both material absorption and scattering from fabrication. Furthermore, we limit our survey to *Q*_i_ in microresonators for integrated nonlinear photonics applications. For instance, while there are reports of high *Q*_i_ TFLN microresonators approaching the material limit of 10^8^^63,64^, they are designed specifically to explore the absorption limits. Instead, we plot the result from Shams-Ansari et al.^54^, with achievable *Q*_i_ up to 5 × 10^6^ in microresonators for applications in integrated nonlinear photonics^65^.

### Fabrication

Taking a wafer as in Fig. 1b, our fabrication process flow includes electron-beam lithography to create a pattern and deposition of alumina to create a hard mask, dry reactive ion etching (RIE) with fluorine, deep RIE for chip separation, and in some cases, annealing for 10 h at 500 °C in air. Our process is amenable to SiO_2_ cladding deposition with an inductively coupled plasma CVD process that offers uniformity, low loss, and low void formation. We explore the use of ma-N resist instead of alumina for pattern transfer to titania-tantala, but the etch selectivity is poor, leading to faceting of the waveguides and incomplete etching of the waveguide and coupler gaps. These imperfections are less evident in devices fabricated using an alumina hard mask.

### Quality factor measurement

We measure microresonator *Q*_i_ for the TE0 mode family, identified by polarization and free-spectral range, over the wavelength range of 1520–1600 nm to sample many modes. We measure transmission across this range and fit each resonance lineshape to a model to extract *Q*_i_^66^, as shown in the inset in Fig. 1d. Here, we set *Q*_c_ such that the coupling parameter *K* ≈ 0.25–0.45 for a consistent measurement across devices with different annealing conditions.

### Absorption measurement

To correct for differences in the microresonators, we scale Δ*λ* by known experimental parameters *λ*_0_, *η*, and *Q* in Fig. 2c. We also correct for facet loss (typically 3–5 dB for each facet) for a more accurate estimate of *I*. We assume that *a* and *k* are the same for all films because they are sputtered by the same process, the microresonators have similar geometries (th = 0.570 μm, RR = 100 μm, RW = 1.5, or 1.6 μm), and they have similar thermo-optic coefficients^41,67^.

### Photorefraction measurement

In Fig. 2e, we measure the steady-state band gap transmission while sweeping a tunable laser across the resonance (solid traces). We fit the band gap mode to extract *ϵ*_0_. We monitor changes to the mode structure that are induced by pumping the PhCR mode with *I* = 100 mW on an oscilloscope. To represent the changes after *t* = 6 minutes, we incorporate Δ*ϵ* into the fit (dashed lines).

## Supplementary information

Supplementary Information PDF

## Data Availability

The data that support the findings of this study are available from the corresponding author upon request.

## Code availability

The code used in this study is available from the corresponding author upon request.
