# Supplementary material for: Amorphous metal oxide mixtures for high-Q integrated nonlinear photonics
Source: Commun Phys. 2026 May 7;9(1):236. doi: 10.1038/s42005-026-02665-w (PMC13364719; doi:10.1038/s42005-026-02665-w)
Supplement: Supplementary file 1 — Supplementary Information PDF [file 42005_2026_2665_MOESM1_ESM.pdf]

Supplementary Information for Amorphous metal oxide mixtures for high-Q integrated nonlinear photonics

Alexa R. Carollo *[1], Atasi Dan[1,2], Haixin Liu[1,2], David R. Carlson[3], Jennifer A. Black †[1], and Scott B. Papp[1,2]

[1] *Time and Frequency Division, National Institute of Standards and Technology, Boulder, CO 80305 USA*
[2] *Department of Physics, University of Colorado, Boulder, CO 80309, USA*
[3] *Octave Photonics, Louisville, CO 80027, USA*

1 Supplementary Note 1: Microresonator dispersion

To satisfy phase-matching conditions for optical parametric oscillation (OPO) and microcomb generation in ordinary ring resonators, we pump devices with anomalous dispersion. For an oxide clad titania-tantala microresonator, we achieve anomalous dispersion with a ring width (RW) = 1.5 µm, ring radius (RR) = 100 µm, and film thickness (th) = 0.800 µm. To characterize the dispersion, we scan a tunable continuous-wave laser across the 1520-1600 nm wavelength range, and measure transmission as a function of wavelength through the microresonator; see Fig. S1a. Because of the geometric and material dispersion, the spacing of the resonance modes is not even across this frequency range. We characterize how the mode spacing changes with the integrated dispersion, $D_{\text{int}} = \nu_\mu - \nu_0 - \text{FSR} \times \mu$, where μ is the mode number relative to the pump mode, ν is the mode frequency, and FSR is the free spectral range; see Fig. S1b. Here, we measure an FSR = 211 GHz. By approximating the mode frequencies as a second-order polynomial, we rewrite $D_{\text{int}} = 1/2 D_2 \mu^2$, where D_2 is related to the group-velocity dispersion (GVD). Our fit gives $D_2 \approx 4$ MHz. Indeed, a positive D_2 indicates anomalous dispersion, validating the nonlinear performance in Fig. 3c in the main text.

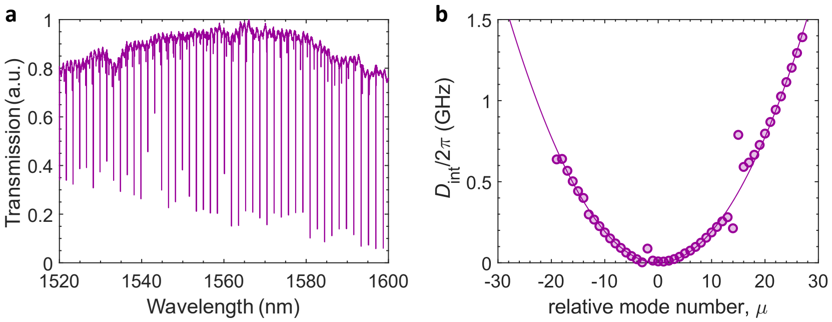

Fig. S1: Microresonator dispersion measurement. (a) Transmission as a function of wavelength in a titania-tantala microresonator. (b) Integrated dispersion, D_{int}, as a function of relative mode number, μ, (points) and its fit with $D_2 = 3.9$ MHz (solid line).

2 Supplementary Note 2: Soliton generation

We confirm that the titania-tantala material supports soliton microcomb generation; see Fig. S2. Here, we test anomalous and normal dispersion photonic crystal microresonators (PhCR), to explore the range of dispersion engineering capabilities of titania-tantala. We perform the experiment as described in "Results and discussion" in the main text, under subsection, "Nonlinearity in titania-tantala integrated devices." We pump devices using an amplified continuous

*alexa.carollo@nist.gov
†Present address: Vescent Photonics, Golden, CO

wave pump laser in the 1550 nm wavelength region, and measure the microcomb spectra using an optical spectrum analyzer (OSA). Indeed, anomalous dispersion allows access to broad spectra (Fig. S2a), while normal dispersion supports dark pulse microcombs (Fig. S2b) with the potential for high efficiency [1, 2].

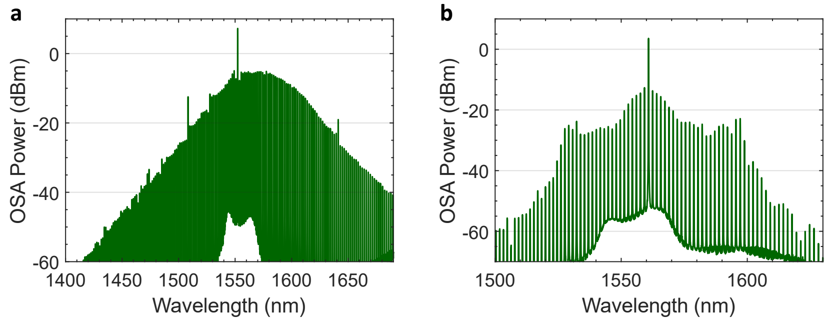

Fig. S2: Soliton generation in titania-tantala photonic crystal microresonators. (a) Microcomb spectrum from an anomalous dispersion device, and (b) microcomb spectrum from a normal dispersion device.

3 Supplementary Note 3: Waveguide dispersion

As an additional test of the nonlinear performance of titania-tantala, we generate supercontinuum spectra in waveguides, as shown in Fig. 3d-f in the main text. For phase matching in waveguides, we design their geometry for anomalous dispersion. To guide our designs, we simulate the group velocity dispersion (GVD) of an oxide clad titania-tantala waveguide with waveguide width (WGW) = 1 µm and film thickness (th) = 0.800 µm; see Fig. S3a. We use finite element analysis to calculate the effective index of the waveguide across the visible and near-IR wavelength regions [3]. Indeed, the dispersion (D) is anomalous in the 1500 nm wavelength region. To understand the supercontinuum spectral properties, we calculate the phase mismatch in Fig. S3b. Our simulation predicts dispersive wave peaks near 750 nm and 2500 nm wavelengths, since at those wavelengths, the phase mismatch is zero [4]. This is consistent with our experiments, where we measure the short-wave dispersive wave peak near 750 nm in the supercontinuum spectrum presented in Fig. 3e in the main text.

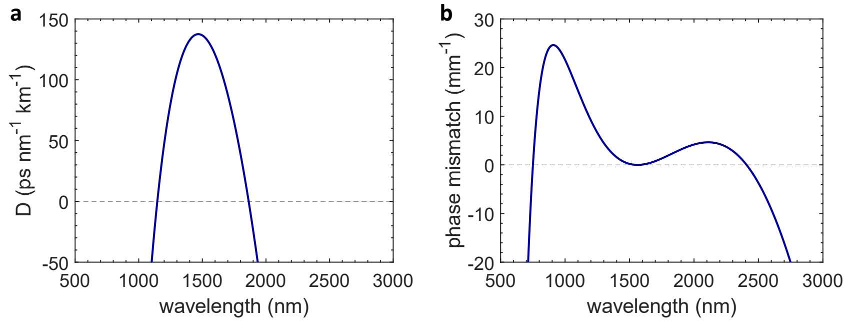

Fig. S3: Waveguide dispersion simulation. (a) Simulated dispersion, D, of a titania-tantala waveguide and (b) corresponding phase mismatch.

Supplementary References

[1] Su-Peng Yu et al. "Spontaneous pulse formation in edgeless photonic crystal resonators". In: *Nature Photonics* 15 (2021), pp. 461–467.

[2] Jizhao Zang et al. "Laser power consumption of soliton formation in a bidirectional Kerr resonator". In: *Nature Photonics* 19 (2025), pp. 510–517.

[3] Kieran F. Lamee et al. “Nanophotonic tantala waveguides for supercontinuum generation pumped at 1560 nm”. In: *Opt. Lett.* 45 (2020), pp. 4192–4195.

[4] Daniel D. Hickstein et al. “Quasi-Phase-Matched Supercontinuum Generation in Photonic Waveguides”. In: *Phys. Rev. Lett.* 120 (2018), p. 053903.
